# Left ventricular myocardial mass index associated with cardiovascular and renal prognosis in IgA nephropathy

**DOI:** 10.1186/s12882-022-02909-1

**Published:** 2022-08-16

**Authors:** Balázs Sági, István Késői, Tibor Vas, Botond Csiky, Judit Nagy, Tibor József Kovács

**Affiliations:** 1 Medical School, Clinical Center, 2Nd. Dep. of Internal Medicine and Nephrology, Diabetes Center, University of Pécs, 1 Pacsirta street, 7624 Pécs, Hungary; 2 Fresenius Medical Care Dialysis Center Pécs, Pecs, Hungary; 3 Department of Internal Medicine Cardiology, Mohács Hospital, Mohács, Hungary

**Keywords:** Cardiovascular risk, Left ventricular hypertrophy, Left ventricular mass index, Chronic kidney disease, IgA nephropathy

## Abstract

**Introduction:**

In chronic kidney disease (CKD), like in IgA nephropathy (IgAN), cardiovascular (CV) mortality and morbidity are many times higher than in the general population, and left ventricular hypertrophy (LVH) is an independent risk factor for CV disease. This follow-up study investigated the association between left ventricular mass index (LVMI) and renal or cardiovascular outcomes.

**Methods:**

We examined 118 IgAN patients prospectively. LVMI and LV geometry was investigated using echocardiography. The primary combined endpoints were total mortality, major CV events, and end-stage renal disease. Secondary endpoints, i.e.—cardiovascular or renal endpoints,—were also examined separately.

**Results:**

Sixty seven percent were males, mean age 53.5 ± 13.5. Mean follow-up time: 184 months. LVMI inversely correlated with eGFR (corr. coefficient: -0.365; *p* < 0.01). We divided the patients into two groups based on the LVMI cut-off suggested by the literature. The presence of LVH caused a worse prognosis in primary (*p* < 0.001), renal endpoints (*p* = 0.01), and also in CV endpoints (*p* = 0.001). The higher LVMI in men significantly worsened the prognosis in all endpoints. Concentric hypertrophy meant a worse prognosis. Independent predictors of LVMI were gender and eGFR in uni- and multivariate regression and hemoglobin levels only in logistic regression. Independent predictors of the primary endpoint were LVMI, eGFR, gender, obesity, HT, DM, and metabolic syndrome in Cox regression analysis.

**Conclusion:**

Increased LVMI may predict the progression to end-stage renal disease and CV events in IgAN. Determining LVMI may be a useful parameter not only in CV risk but also in the stratification of renal risk in CKD.

## Introduction

Chronic kidney disease (CKD) is a leading risk factor for cardiovascular (CV) disease, which reduces patients' life expectancy and causes a significant financial burden [1]. The presence of any degree of renal impairment and/or proteinuria increases the risk for CV disease. Although the mechanism linking CKD to CV disease is not yet fully elucidated, both traditional and non-traditional CV risk factors have been suggested to play a role in it. In this study, we focused on LVH a traditional risk factor, whose presence may be an important cause of the higher incidence of CV cases among patients with CKD. It is known that LVH, determined as LVMI by echocardiography in a non-invasive manner, could be used to predict the risk of cardiovascular mortality among patients with various illnesses, in the whole population [2–6]. IgA nephropathy (IgAN) is the most common immunocomplex-mediated primary glomerular disease all over the world [7]. Patients with IgAN are a relatively homogenous group compared to the whole CKD population. Half of the patient’s kidney disease deteriorated to ESRD within 15 years [7].

The high mortality of CKD patients is primarily due to the high incidence of CV events, of which the development of LVH is a well-known risk factor [8]. LVH is a predictor of all-cause and CV mortality in hypertensive [9] and end-stage renal disease (ESRD) [10]. However, few studies have examined the predictive effect of LVH in patients with earlier stages of CKD (II-IV) [11–14].

Data from a Japanese cross-sectional study (CKD-JAC) published in 2019 revealed that LVH and other risk factors (previous CV disease, blood pressure control, and metabolic status) may also affect renal progression in patients with renal impairment independently from the cause of CKD [15, 16]. Reducing LVMI resulted in decreased mortality in various high-CV risk groups [17]. McQuarrie et al. stated in 2010 that proteinuria is significantly and independently associated with LVMI in patients with CKD [18]. The independent predictors of LVMI and LVH were end-diastolic volume, predialysis systolic BP, and Ca X PO4 in patients on chronic hemodialysis [19].

### Objective

Our study aimed to assess the prognostic significance of LVMI in IgAN. We wished to find out whether elevated LVMI was a predictor of major CV events including myocardial infarction, stroke, revascularization, cardiac death, and ESKD development, independent of other classical risk factors. Subsequently, we also wish to determine the relationship between LVMI and renal function, and the importance of LV geometry change.

### Patients

**Fig. 1:**
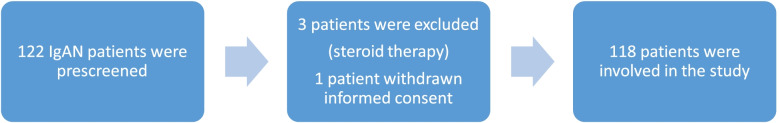
Patients recruitments flow chart

We prescreened 122 patients with renal biopsy confirmed IgAN, 3 patients were excluded due to corticosteroid treatment and 1 patient withdrew her informed consent because she moved to another country. We included 118 patients in the study and we analyzed their data prospectively (Fig. 1). The University of Pécs Regional Research Ethics Committee approved the study protocol, and all participants gave written consent to their completion.

At the start of the patient enrollment, echocardiography measurements were performed and classic CV risk factors including—hypertension, carbohydrate metabolism disorder, obesity, lipid abnormalities, smoking—and patient medication such as antihypertensive drugs (ACEI/ARB, BB, CCB), and statins were also recorded. The obesity inclusion criterion was BMI over 30 kg/m2. Metabolic syndrome was defined according to the ATP III (Adult Treatment Panel III) criteria. The CKD-EPI formula was used to estimate renal function (eGFR, ml/min, 1.73 m^2^). Patients with severe comorbidities (NYHA stage III-IV heart failure, malignancies requiring active treatment) and treated with corticosteroids were excluded. End-stage renal disease (CKD-5) and renal replacement therapy or a history of kidney transplantation were also exclusion criteria. A 24-h blood pressure monitor was used to determine the patient’s 24-h average systolic and diastolic blood pressure, pulse pressure, and diurnal index by Meditech ABPM devices. At the start of the study, echocardiography was performed (see below). Additional CV examinations (ergometry, coronarography, etc.) were also performed based on individual patients’ complaints.

Patients were observed regularly and the follow-up examinations were performed every 3 to 6 months or—more often, when it was necessary. Upon these visits medical events that have taken place since the last visit was registered, physical status was examined, and detailed laboratory tests were conducted. Blood pressure values were determined from the average of 3 measurements taken after 10 min of rest.

The primary composite endpoint of the study consisted of CV outcomes, including total mortality, coronary intervention (due to an acute coronary event or acute myocardial infarction), stroke, and renal outcomes like the development of ESKD. Subsequently, CV and renal endpoints were analyzed separately as secondary endpoints.

### Echocardiographic measurement

Echocardiography was performed with Aloka SSD 1400, two operators were involved in the study. Left ventricular mass (LVM) was calculated from 2D images of the left ventricular short-axis muscle area and apical left ventricular length (LVM = (5/6 area * length)). The assessment of left ventricular mass index (LVMI g/m2) was obtained according to the formula of Devereux; the cardiac mass was indicized also with lean mass. LVMI was determined based on the Cornell criterion and indexed for height (in meters). The left ventricular ejection fraction (LVEF) was calculated by calculating the diastolic and systolic left ventricular volumes using the unidirectional Simpson method: EF = ((Dvol-Svol) / Dvol) * 100. Based on the measured LVMI and relative wall thickness (RWT) values, four different categories of the left ventricle geometry were identified: (1) normal (normal LVMI and normal RWT) (N), (2) concentric remodeling (normal LVMI and increased RWT) (CR), (3) eccentric hypertrophy (elevated LVMI and normal RWT) (EH) and (4) concentric hypertrophy (elevated LVMI and elevated RWT) (CH). Diastolic function was determined by mitral inflow and pulmonary venous flow based on conventional spectral Doppler measurements. We also measured the ratio of the E wave to the A wave (E / A ratio), the isovolumetric relaxation time (IVRT), and the deceleration time of the E wave. LVH was defined as abnormal RWT and/or LVMI.

### Statistical analysis

For statistical analysis, we divided our patients into two groups according to the LVH determined by LVMI (limits were 115 g / m2 for men and 95 g / m2 for women) which is recommended by the literature. All values are mean ± SD unless otherwise indicated. Differences between the two groups were compared by Student’s t-test and Mann–Whitney U test for continuous variables and χ^2^ test for categorical variables. The relationship between two continuous variables was assessed by a bivariate correlation method (Pearson’s correlation). The LVMI influencing factors were examined by uni- and multivariate linear regression analysis. We also used logistic regression uni- and multivariate analysis to assess the associations between LVMI and other covariates. Survival was assessed with the Mantel-Cox log-rank test. The effect of factors influencing survival was analyzed with Cox regression analysis. Data analysis was performed using SPSS version 22.0 (Statistical Package for Social Sciences for Windows, SPSS Inc., Chicago, IL), and *p* < 0.05 was considered statistically significant.

## Results

We included 118 patients with IgAN at the 2^nd^ Department of Internal Medicine Nephrology and Diabetes Center of the Clinical Center at the University of Pécs, who were followed for an average of 184 ± 82 months between 2003 and 2020. The mean age of patients was 53.5 ± 13.5 years, of whom 79 were male. The majority of patients (76%) were hypertensive and 25% were diabetic. The main clinical data and the incidence of risk factors are detailed in Table 1.

**Table 1:** Baseline characteristics of IgAN patients

Clinical data	Patients (*n* = 118)	LVH- (*n* = 27)	LVH + (*n* = 91)	*p* value
Male/Female (n/%)	79/39 (67/33)	13/14 (48/52)	66/25 (73/27)	0.039
Age (year)	53.5 ± 13.5	49 ± 12.9	54.8 ± 13.2	0.023
Average follow-up time (months)	184.2 ± 81.9	200.7 ± 66.4	179.4 ± 85.66	NS
24 h average systolic/diastolic blood pressure (Hgmm)	124/74 ± 13/9	117/70 ± 14/9	126/75 ± 9/7	0.001
24 h pulse pressure (Hgmm)	49.8 ± 9.52	47.2 ± 7.01	50.5 ± 10.03	NS
Diurnal index systolic (%)	9.54 ± 7.01	8.36 ± 5.38	9.88 ± 7.40	NS
Metabolic parameters				
Hypertension (n, %)	90 (76)	14 (52)	76 (83)	0.001
Dyslipidemia (n, %)	54 (46)	9 (33)	45 (49)	NS
Obesity (n, %)	31 (26)	3 (11)	28 (31)	NS
Prediabetes (IFG and IGT) (n, %)	5 (4)	1 (4)	4 (4)	NS
Diabetes mellitus (n, %)	30 (25)	3 (11)	27 (30)	NS
eGFR (ml/min)	85.4 ± 35.8	96.7 ± 39.3	82.0 ± 34.24	0.036
Smoking (n, %)	17 (14)	0 (0)	17 (19)	0.047
Metabolic syndrome (n, %)	27 (23)	2 (7)	25 (27)	0.014
Duration of kidney disease (year)	10.01 ± 9.70	9.40 ± 7.47	10.2 ± 10.30	NS
Echocardiography				
LV EF (%)	62.69 ± 6.39	61.84 ± 6.20	62.93 ± 6.46	NS
LVMI (g/m2)	107.34 ± 23.22	87.08 ± 16.30	113.56 ± 21.51	0.012
LVM (g)	207.66 ± 51.95	165.25 ± 46.03	196.51 ± 46.76	0.022
LVEDD (cm)	4.97 ± 0.43	4.91 ± 0.47	4.99 ± 0.41	NS
DD (%)	55 (47)	9 (32)	46 (51)	NS
E/A	1.05 ± 0.25	1.14 ± 0.29	0.93 ± 0.21	NS
Ergometry				
Average heart rate (beat/min)	73.85 ± 13.92	75.12 ± 8.79	73.75 ± 15.0	NS
Stress test time (s)	575.23 ± 186.73	614.66 ± 195.54	563.53 ± 183.52	NS
CAD (positive stress test)	16 (14)	1 (4)	15 (16)	0.040
Therapy at the start				
ACEI/ARB therapy (n, %)	100 (85)	21 (78)	79 (87)	NS
BB (n, %)	31 (26)	5 (19)	26 (29)	NS
Statin (n, %)	37 (31)	4 (15)	33 (36)	0.027
CCB (n, %)	31 (26)	5 (19)	26 (29)	NS
Laboratory results				
Hb (g/dl)	13.75 ± 1.66	13.93 ± 1.41	13.69 ± 1.73	NS
MAU (mg/day)	466.22 ± 639.65	387.26 ± 629.39	496.89 ± 637.26	NS
UA (umol/l)	321.71 ± 90.57	282.59 ± 88.18	333.02 ± 88.62	NS
Total cholesterol (mmol/l)	4.95 ± 1.24	4.77 ± 0.75	5.01 ± 1.35	NS
HDL chol (mmol/l)	1.28 ± 0.46	1.30 ± 0.35	1.27 ± 0.49	NS
TG (mmol/l)	1.72 ± 1.10	1.36 ± 0.66	1.83 ± 1.18	0.025
Endpoints				
Primary combined endpoints (n/%)	42 (35)	4 (15)	35 (42)	0.028
Cardiovascular events (n/%)	13 (11)	1 (4)	12 (13)	0.045
Renal events (n/%)	29 (24)	3 (11)	26 (28)	0.024

*RR* Blood pressure, *BMI* Body mass index, *eGFR* Estimated glomerular filtration rate, *ACEI* angiotensin-converting enzyme inhibitor, *ARB* Angiotensin receptor blocker, *BB* Beta-blocker, *CCB* Calcium channel blocker, *CAD* Coronary artery disease, *LV EF*, Left ventricle ejection fraction, *LVMI* Left ventricle mass index, *LVM* Left ventricular mass, *LVEDD* Left ventricular end-diastolic diameter, *DD* Diastolic dysfunction, *Hb* Hemoglobin, *MAU* Microalbuminuria, *HUS* Uric acid, *HDL cholesterol High-density lipoprotein cholesterol, TG Triglyceride*

**Fig. 2:**
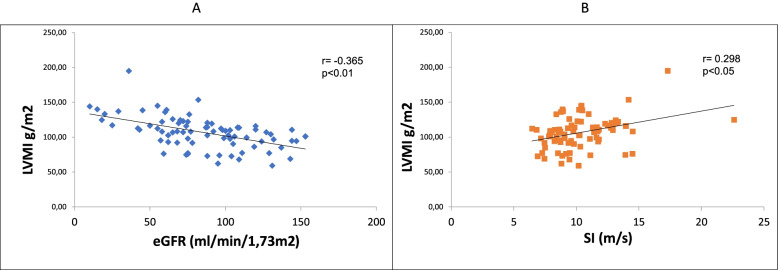
Relationship between LVMI and eGFR

The mean LVMI of the 118 patients was 107.34 ± 23.22 g / m2. Patients were divided into two groups according to the limit for LVH by the LVMI (> 115 g / m2 for men and > 95 g / m2 for women and/or RWT > 0.42) considered abnormal in the literature. Clinical data of patients divided into two groups based on the presence or absence of LVH are listed in Table 1. These two groups of IgAN patients differed significantly in age, gender, the incidence of hypertension, mean blood pressure and renal function, the incidence of metabolic syndrome, statin treatment, and serum triglyceride level. There were no differences between the two groups regarding 24 h pulse pressure, metabolic parameters, dyslipidemia, carbohydrate metabolism, obesity, or using angiotensin-converting enzyme inhibitor (ACEI), or angiotensin II receptor blocker (ARB). LVMI was inversely correlated with eGFR (corr. coefficient: -0.365; *p* < 0.01) (Fig. 2).

When we analyzed 15 parameters by logistic regression analysis: age, obesity, eGFR, hemoglobin, carbohydrate metabolism disorder, and metabolic syndrome were significant influencing factors for LVMI using logistic regression univariate analysis (Table 2).

**Table 2:** Logistic regression univariate analysis of LVMI influencing factors

	UNIVARIATE					
**Parameters**	**B**	**S.E**	**Wald**	**df**	**Sig**	**Exp(B)**
Gender	0.28	0.39	0.51	1	0.475	1.32
Age	0.04	0.02	8.04	1	**0.005**	1.04
Smoking	-0.86	0.55	2.51	1	0.113	0.42
Dyslipidemia	-0.39	0.37	1.10	1	0.295	0.68
Hypertension	-0.81	0.43	3.56	1	0.059	0.45
Obesity	-1.48	0.51	8.5	1	**0.004**	0.23
Carbohydrate metabolism disorder	-1.11	0.44	6.22	1	**0.013**	0.33
Metabolic syndrome	-1.29	0.47	7.43	1	**0.006**	0.28
eGFR	-0.02	0.01	13.93	1	**< 0.001**	0.98
Hb	-0.39	0.12	9.76	1	**0.002**	0.68
MAU	0.0	0.0	0.0	1	0.962	1.0
HUS	0.0	0.0	1.71	1	0.190	1.0
Total cholesterol	-0.01	0.16	0.0	1	0.950	0.99
HDL cholesterol	0.11	0.40	0.08	1	0.777	1.12
TG	-0.17	0.17	0.93	1	0.336	0.85

Model 1: We examined 15 parameters that could influence LVMI

*eGFR* Estimated glomerular filtration rate, *Hb* Hemoglobin, *MAU* Microalbuminuria, *HUS* uric acid, *HDL* cholesterol High-density lipoprotein cholesterol, *TG* Triglyceride

Only hemoglobin level was an independent predictive factor for LVMI using logistic regression multivariate analysis (Table 3).

**Table 3:** Logistic regression multivariate analysis

Parameters	B	S.E	Wald	df	Sig	Exp (B)	95% CI lower	95% CI upper
Age	0.03	0.02	3.31	1	0.069	1.03	1.00	1.07
eGFR	-0.01	0.01	3.08	1	0.079	0.99	0.97	1.00
Hb	-0.33	0.15	5.14	1	**0.023**	0.72	0.54	0.96
Obesity	-0.50	0.61	0.68	1	0.411	0.61	0.18	1.99
Hypertension	-1.23	0.75	2.71	1	0.100	0.29	0.21	1.22
Carbohydrate metabolism disorder	-0.49	1.31	0.14	1	0.708	0.61	0.05	7.92
Metabolic syndrome	-0.50	1.35	0.14	1	0.711	0.61	0.04	8.50

*eGFR* Estimated glomerular filtration rate, *Hb* Hemoglobin

**Fig. 3:**
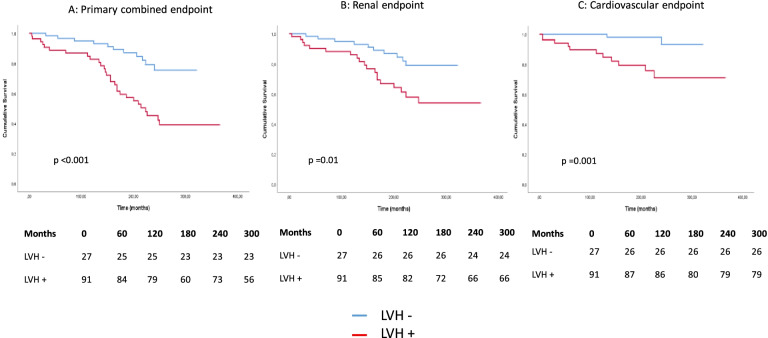
Kaplan–Meier curves in the presence and absence of LVH. (**A**: combined endpoint (renal and CV), **B**: renal endpoint, **C**: CV endpoint)

**Fig. 4:**
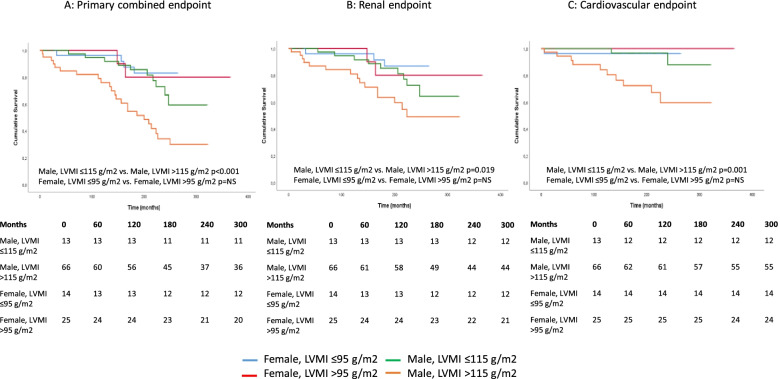
Kaplan–Meier curves based on LVMI in men and women. (**A**: combined endpoint (renal and CV), **B**: renal endpoint, **C**: CV endpoint)

In our follow-up study, the presence of LVH significantly worsened the survival in the case of primary endpoints (*p* < 0.001), renal endpoints (*p* = 0.01), and also in CV endpoints (*p* = 0.001) (Fig. 3). The presence of LVH significantly impaired survival in both primary and secondary endpoints in men; there was no such difference in women (Fig. 4).

**Fig. 5:**
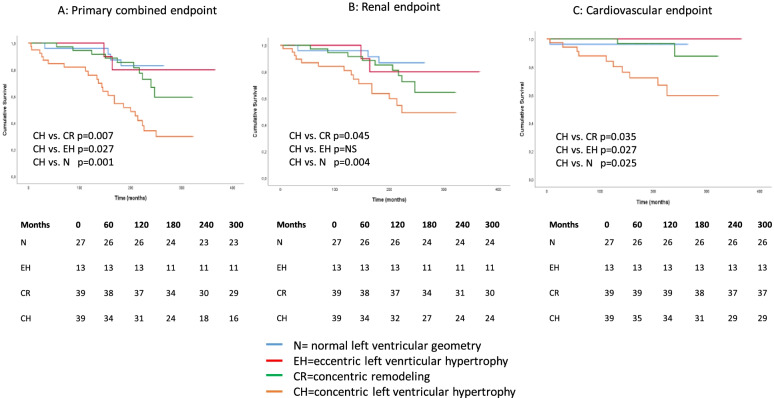
Kaplan–Meier curves for abnormal and normal left ventricular geometry. (**A**: combined endpoint (renal and CV), **B**: renal endpoint, **C**: CV endpoint)

Analyzing the characteristics of LVH on survival, the worst survival was in cases of concentric hypertrophy (*n* = 39) compared to non-hypertrophic patients (LVH- patients, *n* = 27) (CH vs. N; *p* = 0.001), patients with eccentric hypertrophy (*n* = 13) (CH vs. EH; *p* = 0.027 and EH vs N; p = NS) and concentric remodeling (*n* = 39) (CH vs. CR; *p* = 0.007 and EH vs. CR; *p* = NS and CR vs. N; *p* = 0.014) in cases of primary combined endpoints (Fig. 5).

When analyzed by Cox regression analysis, the significant influencing factors for the primary combined endpoint (kidney + CV) were baseline renal function, hypertension, carbohydrate metabolism disorder, obesity, metabolic syndrome, gender, and LVMI, but, dyslipidemia and age were not significant. Significant influencing factors for the secondary renal endpoint were LVMI and eGFR and in the case of secondary CV endpoint LVMI and diabetes—see Table 4.

**Table 4:** Cox regression analysis: primary combined, and secondary renal and cardiovascular endpoints influencing factors

**Parameters**	**B**	**SE**	**Wald**	**df**	**Sig**	**Exp(B)**	**95% CI for Exp(B)** **Lower Upper**	
Primary combined endpoint								
Age	1.419	0.345	4.567	1	0.092	2.136	1.468	2.340
Gender	-0.981	0.418	5.516	1	**0.019**	0.375	0.165	0.850
LVMI	0.036	0.006	34.638	1	**< 0.001**	1.037	1.025	1.050
eGFR	-0.028	0.005	27.322	1	**< 0.001**	0.972	0.962	0.983
Dyslipidemia	0.464	0.326	2.022	1	0.155	1.591	0.839	3.016
Obesity	0.782	0.330	5.626	1	**0.018**	2.187	1.146	4.174
Hypertension	1.845	0.726	6.452	1	**0.011**	6.325	1.524	26.253
Diabetes	1.017	0.323	9.884	1	**0.002**	2.765	1.467	5.212
Metabolic syndrome	1.214	0.324	14.015	1	** < 0.001**	3.366	1.783	6.353
ACEI/ARB therapy	-1.035	1.098	0.889	1	0.346	0.355	0.041	3.055
Renal endpoint								
Dyslipidemia	-0.801	0.508	2.488	1	0.115	0.449	0.166	1.214
Obesity	0.077	0.458	0.028	1	0.866	1.080	0.440	2.651
Hypertension	0.020	0.850	0.001	1	0.981	1.020	0.193	5.393
Diabetes	0.544	0.437	1.548	1	0.213	1.722	0.731	4.054
LVMI	0.024	0.008	9.444	1	**0.002**	1.024	1.009	1.040
eGFR	-0.032	0.008	17.976	1	**< 0.001**	0.968	0.954	0.983
ACEI/ARB	-0.790	1.120	0.498	1	0.481	0.454	0.050	4.077
Cardiovascular endpoint								
Dyslipidemia	0.328	0.693	0.225	1	0.636	1.389	0.357	5.399
Obesity	-0.050	0.630	0.006	1	0.937	0.951	0.277	3.269
Diabetes	1.491	0.631	5.592	1	**0.018**	4.443	1.291	15.296
LVMI	0.046	0.014	11.539	1	**0.001**	1.047	1.020	1.075
eGFR	0.008	0.012	0.483	1	0.487	1.009	0.985	1.033

*LVMI* Left ventricle mass index, *eGFR* estimated glomerular filtration rate, *HRR* Heart rate recovery, *SI* Stiffness index, *DVP* Digital volume pulse, *ACEI* Angiotensin-converting enzyme-inhibitor; *ARB* Angiotensin II-receptor blocker

## Discussion

In our study, we found a correlation between LVMI and renal function in a homogenous immunocomplex-mediated CKD population of IgAN patients. The baseline renal function was an independent predictor of LVMI. In the case of higher LVMI, CV and renal composite endpoints were higher.

It is well known that hypertension is a major factor in the development of LVH in chronic hemodialyzed patients [20]. However, in the early stages of CKD, when blood pressure elevation is not so significant, the mechanism of the relationship between CKD and LVH is not fully characterized.

Echocardiography and ECG have been used to measure LVH in patients with CKD for many years. In patients with advanced renal disease (CKD 3–4), echocardiography may overestimate LVMI due to its dependence on the intravascular volume [21] while ECG, even with definitive criteria, is generally a less reliable method [22]. In recent years, magnetic resonance imaging (CMRI) of the heart has become the gold standard method for measuring left ventricular size as well as the degree of myocardial fibrosis in patients with CKD [23]. However the availability of CMRI is still limited, echocardiography, which is widely used in the daily clinical routine to monitor and follow many patients, is more practical at considerably lower cost, and is a more easily accessible method. Thus the value of the echocardiography examination should be important in CKD.

Previous studies have shown that LVH is more common in patients with CKD than in the general population, affecting 40–78% of patients [24]. In addition, there is a gradual increase in LVMI with the progression of CKD [24, 25]. CKD and CVD share risk factors such as hypertension, vascular stiffness, and endothelial dysfunction [26, 27]. The pathomechanism of increasing LVMI during CKD is unknown, although we also observed here an inverse relationship between LVMI and renal function. Thus it seems important to follow up on the changes in LVMI.

Known CV risk factors such as baseline eGFR, proteinuria, and hypertension are also risk factors for CKD progression, contributing to the acceleration of renal function loss and progression to ESKD. However, the progression of CKD, which is a complex process, cannot be explained in all cases by these traditional risk factors.

In our study, we analyzed the associated factors of LVMI and the predictive effect of LVMI for CV and renal endpoints. Based on the correlation between LVMI and GFR in IgAN, elevated LVMI may predict the progression of renal disease and CV events, especially in men, before reaching ESKD. In our IgAN patients, we found that the increased LVMI had a significant effect on both combined and renal and CV outcomes in males. This can be partly explained by the increased incidence of male CV events in general. Male IgAN patients had worse progression for CKD, in part due to more severe CV complications as known from an earlier study published by Deng et al. [28].

Paoletti et al. observed similar results in non-homogeneous chronic kidney patients with higher LVMI than non-diabetic CKD patients in stages 3–4. In patients with stage 1 CKD, it was proved that LVMI is a good prognostic indicator of mortality [29]. A similar finding was obtained by Huang et al. showing that patients with higher LVMI had a higher risk of impaired renal function regardless of the degree of renal impairment [30].

By contrast, Tripepi et al. measured the risk of death and heart failure in CKD (CRIC study) and found that LVMI alone did not provide a clear prognostic value. The discrepancy between the studies may be explained by the fact that the patients of the CRIC study were not a homogeneous CKD population and included CKD patients with different etiologies, follow-up time was shorter and the endpoints were different (only death and de novo heart failure). In our study, we examined a homogeneous patient population with IgA nephropathy [31], and with longer follow-up, and, despite a lower number of cases, we were able to strengthen the prognostic role of LVH.

Eckardt et al. examined the type of LVH in CKD. They found that the prognosis was the worst in those with eccentric LVH and intermediate in those with concentric LVH [32]. Paleotti et al. found that LVH is a strong predictor of the risk of poor CV and renal outcomes independent from LV geometry in patients with CKD [33]. In contrast to these studies, we found that the presence of concentric hypertrophy in IgAN patients was the worst prognostic LV geometry in both sexes. Other studies found concentric hypertrophy the most common LV geometry alteration and the worst in terms of progression in general, in the case of hypertension, obesity, sleep apnea syndrome, and CV disease [34, 35]. Our data suggest that, like in other diseases, concentric hypertrophy is the worst prognosis in IgAN (and possibly in other CKDs).

We suggest that an increased LVMI and a decreased eGFR may synergistically have an impact on the poor prognosis. Worsening renal function, higher incidence of LVH, and CV complications resulting in a worse prognosis and impaired left ventricular geometry (higher LVMI). As a result of this, LVH and impaired renal function seem to be enhancing processes for each other. But the connection between the deterioration of kidney function and LVH is not elucidated.

Animal and human studies showed that elevated FGF23 levels in CKD can induce the development of LVH. It has also been confirmed that activation of local RAAS via the FGF23-mediated process promotes myocardial hypertrophy and fibrosis [36]. So, these non-specific alterations in CKD may play a role in IgAN as well. Recent biomarker studies have consistently highlighted the importance of LVH in the progression of CKD. In addition to echocardiography, the measurement of these biomarkers may provide additional data for screening high-risk patients to initiate early treatment. In the MESA study [37], cardiac MR-defined “malignant” LVH, which refers to the co-existence of LVH and elevated soluble cardiac biomarkers (such as troponin T for myocardial damage, NT-proBNP for myocardial hemodynamic stress) and LVH, may predict asymptomatic left ventricular dysfunction, the development of heart failure (particularly the HFrEF), and CV mortality in the general population. It also draws attention to the need for more aggressive treatment of these patients with “malignant” LVH. Among patients with CKD, a novel biomarker, so-called growth differentiation factor 15 (GDF-15) was associated with abnormal left ventricular structure and early changes in left ventricular function measured by echocardiography [38].

Some studies examined different biomarkers in the pathomechanism of LVH in CKD. Kim et al. [39] in CKD patients demonstrated that serum Klotho is an independent biomarker of LVMI but it was not that of arterial vessel wall stiffness. Protein-bound uremic toxins (such as indoxyl sulfate (IXS) and p-cresol sulfate (PCS), which have been described in recent years, may also have a pathogenic role in the development of LVH, and asymptomatic cardiac dysfunction due to their cardiotoxic effects [40], although these effects are not specific for IgAN, but rather for CKD. Further examinations are needed to find the most important biomarkers in the pathomechanism of LVH in CKD.

Increased RAAS activity and hypertension in CKD also increase the incidence of vascular events thus RAAS blockade is the standard treatment (recommended in all guidelines) in these patients in general and also for the patients who have IgAN [41–47]. Based on our former results, and others, we thought that RAAS also plays a key role in the development of arterial stiffness and LVH in renal disease as in IgAN [42–44]. In our study, 76% of the IgAN patients were hypertensive, similarly to those in the study by Wanga et al. (71% hypertensive of IgAN patients [41]. Hypertension is a very common complication in IgAN [41, 42] affecting 50–70% of patients, and this was confirmed by our data. However, there are no data on whether ACEI and/or ARB treatment could promote LVMI-lowering effects in patients with IgAN. In our study, at the start more than 75% of the patients received ACEI and/or ARB therapy, therefore, we were not able to analyze users and non-users of RAAS inhibitor treatment. By the end of the observation, almost all patients received RAAS blockers.

In our study, there was no significant difference in the use of a RAAS inhibitor between LVH and non-LVH patients. Based on this observation, RAAS may not be so important in the evaluation of LVH. However, it should be noted that the blood pressure of the study population was well treated. Patients with IgAN exhibiting higher LVMI had a deteriorated renal function, and increased incidence of ESKD and CV complications in both sexes, compared to those with lower myocardial mass. However, this may be particularly important, since further progression of CKD may be accelerated in older age and with impaired renal function. More complications may develop with worse CV status in these patients, which may also worsen the prognosis by creating a “vicious circle”.

Nohara et al. described that in early CKD (stages 1–3) patients, LVMI, urinary protein, and Hb levels were independently associated with factors for progression to dialysis [48]. Similarly in our cohort, there was also an independent association between LVMI and Hb, but not with urinary protein. These also highlight the need to treat hypertension and anemia to prevent LV remodeling not only during the dialysis stage but also from early CKD stages [49]. In CKD patients, there is a better survival rate among those treated with EPO up to a hemoglobin level of 10–12 g/dl, whereas normalization of hemoglobin levels was not beneficial [50]. In our study, there was no significant difference between the lower and higher LVMI group in Hb level, and ESA was used in neither group.

In the latest meta-analysis by Maki et al., it was shown that LVM change may be useful as a surrogate marker the for benefits of interventions intended to reduce mortality risk in CKD [51].

It is conceivable that autoimmune pathways are activated in IgAN, and low-level inflammation can also contribute to the development of LVH and the increase of LVMI, so in IgAN, not only the deterioration of kidney function can lead to the development of LVH. In addition to all this, the endothelial damaging effect of autoimmune processes can also play a role in this. The type of disease can be a predictor or an aggravating factor of LVH in certain cases. Therefore, there may be differences in the development of LVH, for example, between IgAN and polycystic kidney disease. The common point of connection may be differences in the frequency of occurrence of hypertension and subtypes of LVH (eccentric or concentric hypertrophy). Further studies are needed to find out which type of LVH means a worse prognosis for which disease*.*

### Limitations of the study

Our results indicated that LVMI the value obtained from echocardiography has prognostic significance, however, difficulties may occur during echocardiographic measurement. In some subpopulations, specifically the elderly, a lack of cooperation can be a problem. Renal function was determined by estimating GFR, which is widely accepted in the literature. The extent and change of proteinuria were not examined in the present study. The evaluation of the results may also be weakened by the low number of cases and especially the low number of female patients. To exclude interindividual differences, in the echocardiography exam two investigators examined all patients. We did not examine the histological abnormalities underlying progression, such as microvascular damage (TMA/MA = thrombotic microangiopathy/microangiopathy) in the kidney biopsy specimens. We did not focus on the changes in the LVMI in our cohort, although it would also have been an important parameter for prognosis.

Despite these limitations, the results of this study highlight that the onset of target organ damage in CKD is predicted by increased LVH.

## Conclusion

Our results suggest that LVMI assessed by echocardiography appears suitable for setting an estimated prognosis in IgAN patients, the higher LVMI could be an independent prognostic factor for ESKD and CV events. Higher LVMI should call attention to those CKD patients who have higher renal and CV risk earlier stage of CKD (II-IV) and need to be monitored more closely, referred for further CV tests, and given maximal renal and heart protection.

Our findings support the role of echocardiography in the high-CV risk population of CKD patients which also helps to understand the relationship between structural heart abnormalities and renal impairment. Based on all these seems concentric LV hypertrophy should be given special attention.

In conclusion, impaired renal function gradually correlates with LVH in patients with IgAN, and there is also a strong relationship between myocardial mass and eGFR. Decreased renal function is associated with increased LVMI which is responsible for a poorer prognosis due to worse CV and renal outcomes. In the background, the role of common renal and myocardial pathological remodeling could be hypothesized.

To confirm our results, further large-scale, multicenter, prospective studies are warranted to evaluate the role of CV risk factors in mediating the changes of the left ventricular geometry, as well as the complex relationships between CV disease and CKD.

## Data Availability

The datasets used and analyzed during the current study are available from the corresponding author on reasonable request.

## Abbreviations

ACEI: Angiotensin-converting enzyme-inhibitor
ARB: Angiotensin-II-receptor blocker
ATP: Adult treatment panel
BB: Beta-blocker
BMI: Body mass index
CAD: Coronary artery disease
CCB: Calcium channel blocker
CKD: Chronic kidney disease
CH: Concentric hypertrophy
CR: Concentric remodeling
CV: Cardiovascular
CVD: Cardiovascular disease
CMRI: Cardiac magnetic resonance imaging
DD: Diastolic dysfunction
eGFR: Eccentric hypertrophy
ESA: Erythropoietin stimulating agent
ESKD: End-stage kidney disease
FGF23: Fibroblast growth factor 23
Hb: Hemoglobin
HDL: High-density lipoprotein
HFrEF: Heart failure with a reduced ejection fraction
HUS: Uric acid
HRR: Heart rate recovery
IgAN: IgA nephropathy
IVRT: Isovolumetric relaxation time
IXS: Indoxyl-sulfate
LDL: Low-density lipoprotein
LVEDD: Left ventricle end-diastolic diameter
LVEF: Left ventricular ejection fraction
LVH: Left ventricular hypertrophy
LVM: Left ventricular mass
LVMI: Left ventricular mass index
MAU: Microalbuminuria
NYHA: New York Heart Association
NT-proBNP: N-terminal pro B-type natriuretic peptide
PCS: P-cresol-sulphate
RAAS: Renin–angiotensin–aldosterone system
BP: Blood pressure
RWT: Relative wall thickness
TG: Triglyceride
